# Outbreak of Sudden Death with Acute Encephalitis Syndrome Among Children Associated with Exposure to Lychee Orchards in Northern Bangladesh, 2012

**DOI:** 10.4269/ajtmh.16-0856

**Published:** 2017-07-24

**Authors:** Mohammed Saiful Islam, Ahmad Raihan Sharif, Hossain M. S. Sazzad, A. K. M. Dawlat Khan, Murshid Hasan, Shirina Akter, Mahmudur Rahman, Stephen P. Luby, James D. Heffelfinger, Emily S. Gurley

**Affiliations:** 1 International Centre for Diarrheal Diseases Research (icddr,b), Dhaka, Bangladesh;; 2 Institute of Epidemiology Disease Control and Research (IEDCR), Dhaka, Bangladesh;; 3 Centers for Disease Control and Prevention (CDC), Atlanta, Georgia;; 4 Center for Innovation in Global Health, Stanford University, Stanford, California

## Abstract

Recurrent outbreaks of acute encephalitis syndrome (AES) among children in lychee growing areas in Asia highlight the need to better understand the etiology and the context. We conducted a mixed-methods study to identify risk factors for disease, and behaviors and practices around lychee cultivation in an AES outbreak community in northern Bangladesh in 2012. The outbreak affected 14 children; 13 died. The major symptoms included unconsciousness, convulsion, excessive sweating, and frothy discharge. The median time from illness onset to unconsciousness was 2.5 hours. The outbreak corresponded with lychee harvesting season. Multiple pesticides including some banned in Bangladesh were frequently used in the orchards. Visiting a lychee orchard within 24 hours before onset (age-adjusted odds ratio [aOR] = 11.6 [1.02–109.8]) and 3 days (aOR = 7.2 [1.4–37.6]), and family members working in a lychee orchard (aOR = 7.2 [1.7–29.4]) and visiting any garden while pesticides were being applied (aOR = 4.9 [1.0–19.4]) in 3 days preceding illness onset were associated with illness in nearby village analysis. In neighborhood analysis, visiting an orchard that used pesticides (aOR = 8.4 [1.4–49.9]) within 3 days preceding illness onset was associated with illness. Eating lychees was not associated with illness in the case–control study. The outbreak was linked to lychee orchard exposures where agrochemicals were routinely used, but not to consumption of lychees. Lack of acute specimens was a major limitation. Future studies should target collection of environmental and food samples, acute specimens, and rigorous assessment of community use of pesticides to determine etiology.

## INTRODUCTION

Acute encephalitis syndrome (AES) affects approximately 133,000 children each year in Asia.^1–3^ Several different viruses, bacteria, fungus, parasites, chemicals, and toxins can cause AES.^4–6^ Although data addressing the cause of AES in Asia are limited, some studies have identified Japanese encephalitis virus (JEV) and herpes viruses as the most common causes in south Asia, though these etiologies explain only a small fraction of illness.^7–11^ Over the past decades, unusual outbreaks of AES associated with severe illness and death among children have repeatedly been reported from India, Bangladesh, Vietnam, and Thailand.^11–14^ Based on clinical, laboratory, and environmental findings, investigators have speculated that the cause of some of the illnesses in these AES outbreaks may not be infectious; however, no conclusive evidence about etiology has been published following these investigations.^6,15^

In India, outbreaks of AES among children have been reported near lychee orchards almost every year in the Muzaffarpur District of Bihar State since 1995.^6^ The outbreaks generally peak in June and continue until July, corresponding to the lychee harvesting period, which led to assertions of lychee or lychee orchard–associated AES.^6,12,15–17^ Studies conducted in 2013 and 2014 in Muzaffarpur found that AES case-patients had spent more time in agricultural fields or lychee orchards compared with hospital controls and investigators argued that the AES in children could be caused by a toxin, such as methylenecyclopropyl-glycine in lychees, which can lead to hypoglycemia.^6,16,17^ In 2014, a team from the National Vector Borne Disease Control Program in India found concentrations above minimum limits for humans of alpha cyphermethrin, a highly active pyrethroid insecticide, in lychee samples collected from AES outbreak–affected areas and suspected there might be a link between AES outbreaks and pesticide poisoning.^5^ Investigators in Vietnam also found a spatiotemporal association between AES and lychee cultivation in Bac Giang Province from January 2004 to December 2009 and hypothesized a wide range of causes including viral encephalitis, vector borne diseases, and toxic contamination of lychees.^12^ Between 2008 and 2016, icddr,b and Institute of Epidemiology, Disease Control and Research (IEDCR) detected and investigated nine AES outbreaks among children in Bangladesh, of which seven outbreaks occurred during the lychee harvesting season and two outbreaks were near lychee orchards.^18^

On June 16, 2012, a physician from Dinajpur Medical College Hospital notified the IEDCR, the Ministry of Health and Family Welfare, Government of Bangladesh, about an outbreak of encephalitis among children in northern Bangladesh. A conventional epidemiological study focuses on identifying the proximate, individual-level risk factors of the outbreak that often ignores the context in which the outbreak occurred.^19^ To understand an outbreak thoroughly enough to devise prevention strategies, we often require individual-level risk exposures along with the factors that contributed the outbreak to occur.^20^ Therefore, a collaborative team of clinicians, epidemiologists, and social scientists from IEDCR and icddr,b investigated the outbreak. The epidemiologists focused on describing the clinical presentation and identifying proximate individual-level risk factors and the social scientists explored behaviors and practices in affected communities that may have contributed to the outbreak.

## METHODS

### Case definition, case detection, and data collection.

The team conducted the study between June 17 and September 20, 2012. The investigation team defined a case-patient as any child aged 1–12 years who had been admitted to Dinajpur Medical College Hospital with either unconsciousness or convulsions, with or without fever, from May 31 to June 30, 2012. The team reviewed admission registers in the pediatric ward, identified case-patients, and collected addresses of case-patients and preliminary information about signs and symptoms from the physicians. Social scientists visited all case-patient households and used qualitative research techniques to build rapport with and better understand the community perspective about the illnesses.^21^ The team conducted 14 in-depth interviews with case-patient family caregivers, to inquire about the children’s exposures prior to illness onset, chronological order of symptom development, and sequence of health-seeking behaviors. From the preliminary assessment, the team identified that some of the affected case-households were adjacent to lychee orchards. Therefore, the team conducted unstructured observations of all the case-households and their surroundings to understand the proximity to and contact with lychee orchards and any use of pesticides in those orchards. Social scientists also conducted 12 in-depth interviews with neighbors of case-patients and lychee orchard caretakers in the community, inquiring about the frequency and types of pesticides used in or around case-households, in the lychee orchards, and case-patient exposures to areas where pesticides were recently used. This information helped us to generate the hypothesis that exposure to toxic chemicals might have caused illness among children and that was tested using a case–control study.

### Case–control study.

After the qualitative investigation and preliminary data analysis, the investigation team returned to the outbreak-affected communities to conduct a case–control study from September 15 to 20, 2012, to investigate risk factors for disease in the affected areas. Cases spanned a wide geographic area and we hypothesized that there might be something special about the case-patient communities that led to the illness. Therefore, we enrolled two sets of controls: neighborhood controls and nearby village-level controls. Neighborhood controls were selected from the case-patient’s neighborhood, defined as the neighboring house closest to the case-patient household. We anticipated that the controls within the village would help us identify exposures in the neighborhood that led to the illness and the controls from nearby villages would help us identify village-level behaviors and practices associated with the disease. We selected four age-matched neighborhood controls for each case-patient by identifying the four closest courtyards to the case-patients’ courtyard, and enrolling the child closest in age to the case as a control. The team selected one control from each courtyard and the selection was repeated at the next closest household until we enrolled four controls for each case-patient. Eligible controls were those children who had not experienced any illness between May 30 and June 30, 2012, and were aged ±1 year if the case was ≤ 5 years old and ±2 years if the case was between 6 and 12 years old. If there were no children in the selected household that matched our selection criteria, then we selected children from the next closest household. Village-level controls were selected from the nearby villages closest to the case-patient village that did not have any cases identified between May 30 and June 30, 2012. To select village-level controls, the team visited the center of each control village, rolled a pen on the ground, and selected the first household in the direction that the pen pointed. Following the same method described above, we also selected four age-matched village-level controls for each case-patient. We used proxy respondents for all case-patients and controls as all were children aged less than 10 years. Proxy respondents were family members or orchard caretakers who were aware of the case-patients activities and probable risk exposures during May 25, 2012 and June 23, 2012. We used multiple proxy respondents to maximize reliability of the information as family members were more likely to be informed about possible domestic exposures, whereas orchard caretakers were likely to be better informed about orchard exposures.

Based on the preliminary qualitative findings, we developed a structured questionnaire in Bengali language and interviewed the family members of the case-patients and their age-matched controls about their food and environmental exposures in the 3 days preceding the case-patients’ illnesses.

### Data analysis.

We described case-patients in terms of demographics and clinical profiles. We recoded ‘‘do not know’’ responses as ‘‘missing’’ and “not applicable” as “no” during the case–control analysis. Since differences in recall are common between cases and controls in case–control studies, we compared the proportion of cases with missing exposure information to the proportion missing among controls. We estimated age-adjusted odds ratio (aOR) as a measure of association with 95% confidence intervals (CI) between each exposure and illness development using logistic regression.^22^

The qualitative team reviewed the observation and interview field notes, expanded the jottings, and summarized them. The primary author (Mohammed Saiful Islam) read summaries of the observations and interviews, identified themes, and categorized the data according to these themes.^23^

### Ethical considerations.

The team obtained verbal informed consent from study participants. The study protocol was reviewed by IEDCR and was determined to be an urgent public health activity for disease control and not a research activity requiring formal human subjects review.

## RESULTS

**Figure 1.**
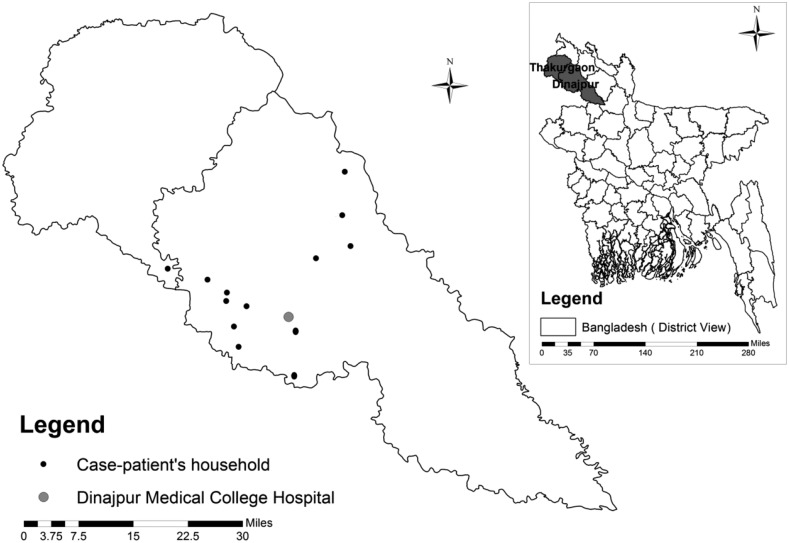
Location of the case-patients’ households and the Dinajpur Medical College Hospital, 2012.

Fourteen patients met our case definition (Table 1). Thirteen were identified from 13 different villages of four subdistricts in Dinajpur District. One was identified from a village of neighboring subdistrict in Thakurgaon District (Figure 1). The median age of case-patients was 4.6 years (IQR = 2.5–5.0 years). The onset of illness of the case-patients was distributed during the day time and night time. Illness started with a sudden outcry among 43% of the case-patients that awakened the patient and their family members from sleep. Family caregivers reported that 79% of case-patients had convulsions. Fifty-seven percent of case-patients had excessive sweating and 50% had frothy discharge from the mouth. Ninety-three percent of case-patients became unconscious; the median time from onset of symptoms or signs to unconsciousness for the six cases for whom information was available was 2.5 hours (Table 1). In the hospital, we found medical records for nine case-patients; four records mentioned that patients had mid-dilated or fixed pupils and six mentioned that patients had lung crepitations on auscultation. Before seeking care at the tertiary care hospital, 64% of case-patients reportedly sought care from spiritual healers, 57% from village doctors and paramedics, and 29% from subdistrict-level hospitals.

**Table 1:** Sociodemographic and clinical profiles of the case-patients of lychee outbreaks in northern Bangladesh, 2012

Characteristics	No. of observation	*N* (%)
Age		
Mean in years	14	4.7
Male	14	9 (64)
Clinical features		
Loss of consciousness	14	13 (93)
Convulsion	14	11 (79)
Lung crepitations on auscultation	9*	6 (67)
Excessive sweating	14	8 (57)
Excessive respiratory secretions	14	7 (50)
Frothy discharge from mouth	14	7 (50)
Generalized weakness/paralysis	14	5 (47)
Fixed dilated pupils	14	4 (44)
Sudden cry	14	6 (43)
Fever	14	4 (29)
Vomiting	14	4 (29)
Urinary incontinence	14	3 (21)
Cold skin	14	3 (21)
Difficulty breathing	14	3 (21)
Diarrhea	14	2 (14)
Unexplained lacrimation	14	1 (7)
Median time from onset of illness to unconsciousness	6†	2.5 hours
Median time from onset of illness to death	13‡	20 hours

* Medical records were available for nine case-patients.

† Among the 13 case-patents who went on unconscious, information were available from six case-patients.

‡ One case-patient survived.

### Case–control study.

We enrolled 14 cases, 56 neighborhood controls, and 56 nearby village-level controls. Mean age (±standard deviation) of the case-patients was 4.7 ± 2.6 years and of the controls was 4.7 ± 2.5 years. In the village-level case–control analysis, cases were 11.6 times (91% versus 54%, aOR = 11.6, 95% CI = 1.02–109.8) more likely to have visited a lychee orchard in the 24 hours and 7.2 times (85% versus 46%, aOR = 7.2, 95% CI = 1.4–37.6) more likely to have visited a lychee orchard in the 3 days preceding illness onset compared with the controls from the nearby villages using logistic regression (Table 2). Moreover, case-patient family members were 7.2 times (43% versus 9%, aOR = 7.2, 95% CI = 1.7–29.43) more likely to work in a lychee orchard than the family members of controls from the nearby villages. A similar pattern of elevated odds ratios was also found in the neighborhood control analysis. Cases were 4.1 times (85% versus 59%, aOR = 4.1, 95% CI = 0.8–21.8) more likely to have visited a lychee orchard in the 3 days preceding illness onset compared with the controls from the neighboring households. Family members of case-patients were also 1.8 times (43% versus 30%, aOR = 1.8, 95% CI = 0.5–5.9) more likely to work in a lychee orchard than the family members of controls from the neighboring household were, though these associations were not statistically significant (Table 3).

**Table 2:** Exposures associated with illness among cases and nearby village controls using logistic regression, northern Bangladesh, 2012

Exposures	Case*	Control*	aOR†	*P* value
	*n*/*N* (%)	*n*/*N* (%)	(95% CI)	
Exposures in the 24 hours preceding illness onset of case-patients				
Food				
Tube well water	13/14 (93)	55/55 (100)	1	Undefined
Lychee	8/14 (57)	22/31 (71)	0.6 (0.1–2.1)	0.39
Mango	6/14 (43)	22/26 (85)	0.1 (0.0–0.6)	0.00
Papaya	0/14 (0)	1/35 (3)	1	Undefined
Potato	11/14 (79)	39/42 (93)	0.3 (0.0–1.6)	0.15
Ladies finger	1/14 (3)	4/28 (14)	0.4 (0.0–4.4)	0.49
Egg plant	4/14 (29)	2/20 (10)	3.8 (0.5–28)	0.18
Tomato	1/14 (7)	1/28 (4)	1.6 (0.1–31)	0.75
Banana	1/14 (7)	3/26 (12)	0.6 (0.1–6.4)	0.67
Bitter gourd	0/14 (0)	1/32 (3)	1	Undefined
Corn	0/13 (0)	1/35 (3)	1	Undefined
Lentils	1/14 (7)	7/22 (32)	0.1 (0.0–1.5)	0.11
Environment				
Visiting lychee orchard	10/11 (91)	20/37 (54)	11.6 (1.02–109.8)	0.03
Visiting a mango orchard	9/13 (69)	24/40 (60)	1.5 (0.4–5.7)	0.55
Visiting a vegetable garden	3/14 (21)	8/54 (15)	1.5 (0.3–6.8)	0.58
Visiting an orchard that leased for commercial production	9/14 (64)	23/55 (42)	2.6 (0.8–8.9)	0.13
Exposures in the 3 days preceding illness onset of case-patients
Visiting a lychee orchard	11/13 (85)	23/50 (46)	7.2 (1.4–37.6)	0.02
Visiting a mango orchard	8/13 (62)	26/48 (54)	1.4 (0.4–4.7)	0.63
Visiting a vegetable garden	3/14 (21)	10/50 (20)	1.2 (0.2–4.5)	0.93
Visiting any garden that used pesticides	4/14 (29)	6/51 (12)	3.2 (0.7–14.4)	0.11
Plucked lychees from the orchard	2/13 (15)	12/53 (23)	0.6 (0.01–3.01)	0.56
Plucked mangoes from the orchard	1/14 (7)	4/54 (7)	0.98 (0.1–10.4)	0.98
Visiting any garden while pesticides were being applied	5/13 (38)	6/43 (14)	4.9 (1.0–19.4)	0.05
Other exposures				
Having lychee orchard adjacent to the house	13/14 (93)	38/56 (68)	6.6 (0.8–6)	0.08
Having mango orchard adjacent to the house	11/14 (79)	39/56 (70)	1.6 (0.4–6.7)	0.49
Having vegetable garden adjacent to the house	7/13 (54)	31/54 (57)	0.8 (0.2–2.8)	0.76
Family members work in lychee orchard	6/14 (43)	5/53 (9)	7.2 (1.7–29.4)	0.01
Family members work in mango orchard	1/14 (7)	0/54 (0)	1	Undefined
Family members work in vegetable garden	0/14 (0)	2/55 (4)	1	Undefined
Family members with occupation in agriculture	11/13 (85)	35/51 (69)	2.5 (0.5–12.7)	0.26
History of animal or insect bite	1/14 (7)	1/54 (2)	4.3 (0.2–76.1)	0.24

aOR = age-adjusted odds ratio; CI = confidence interval.

* Family members were not aware of some exposures of the cases and controls. Denominator has been changed due to recoding “do not know” into “missing value.”

† Age adjusted odds ratio.

**Table 3:** Exposures associated with illness among cases and neighborhood controls using logistic regression, northern Bangladesh, 2012

Exposures	Case*	Control*	aOR†	*P* value
	*n*/*N* (%)	*n*/*N* (%)	(95% CI)	
Exposures in the 24 hours preceding illness onset of case-patients				
Food				
Tube well water	13/14 (93)	54/56 (96)	0.5 (0.04–5.8)	0.57
Lychee	8/14 (57)	27/44 (61)	0.9 (0.2–2.9)	0.82
Mango	6/14 (43)	24/41 (59)	0.5 (0.1–1.8)	0.31
Papaya	0/14 (0)	0/40 (0)	1	Undefined
Potato	11/14 (79)	43/45 (96)	0.2 (0.0–1.1)	0.06
Ladies finger	1/14 (7)	6/38 (16)	0.4 (0.04–3.8)	0.44
Egg plant	4/14 (29)	5/39 (13)	2.7 (0.6–12.1)	0.18
Tomato	1/14 (7)	6/41 (15)	0.4 (0.04–4.2)	0.48
Banana	1/14 (7)	4/40 (10)	0.7 (0.07–7.08)	0.77
Bitter gourd	0/14 (0)	2/43 (5)	1	Undefined
Corn	0/13 (0)	5/42 (12)	1	Undefined
Lentils	1/14 (7)	5/38 (13)	0.5 (0.1–4.7)	0.55
Environment				
Visiting lychee orchard	10/11 (91)	31/37 (84)	2.3 (0.2–25.0)	0.47
Visiting a mango orchard	9/13 (69)	27/44 (61)	1.5 (0.4–5.7)	0.56
Visiting a vegetable garden	3/14 (21)	11/54 (20)	1.6 (0.2–4.5)	0.92
Visiting an orchard that leased for commercial production	9/14 (64)	33/54 (61)	1.2 (0.32–4.1)	0.81
Exposures in the 3 days preceding illness onset of case-patients				
Visiting a lychee orchard	11/13 (85)	33/56 (59)	4.1 (0.8–21.8)	0.09
Visiting a mango orchard	8/13 (62)	30/54 (56)	1.3 (0.4–4.7)	0.66
Visiting a vegetable garden	3/14 (21)	15/47 (32)	0.6 (0.1–2.3)	0.44
Visiting any garden that used pesticides	4/14 (29)	3/56 (5)	8.4 (1.4–49.9)	0.02
Plucked lychees from the orchard	2/13 (15)	17/55 (31)	0.3 (0.1–2.0)	0.21
Plucked mangoes from the orchard	1/14 (7)	4/56 (7)	1.0 (0.1–9.8)	0.99
Visiting any garden while pesticides were being applied	5/13 (39)	10/50 (20)	2.5 (0.6–9.5)	0.17
Other exposures				
Having lychee orchard adjacent to the house	13/14 (93)	51/56 (91)	1.3 (0.1–11.9)	0.83
Having mango orchard adjacent to the house	11/14 (79)	41/56 (73)	1.4 (0.3–6.1)	0.65
Having vegetable garden adjacent to the house	7/13 (54)	31/55 (56)	0.8 (0.2–2.9)	0.76
Family members work in lychee orchard	6/14 (43)	16/54 (30)	1.8 (0.5–5.9)	0.35
Family members work in mango orchard	1/14 (7)	3/56 (5)	1.4 (0.1–14.3)	0.79
Family members work in vegetable garden	0/14 (0)	5/54 (9)	1	Undefined
Family members with occupation in agriculture	11/13 (85)	39/53 (74)	2.01 (0.4–10.4)	0.40
History of animal or insect bite	1/14 (7)	1/54 (2)	4.2 (0.2–74.1)	0.32

aOR = age-adjusted odds ratio; CI = confidence interval.

* Family members were not aware of some exposures of the cases and controls. Denominator has been changed due to recoding “do not know” into “missing value.”

† Age adjusted odds ratio.

In the village-level case–control analysis, cases were 4.9 times (38% versus 14%, aOR = 4.9, 95% CI = 1.0–19.4) more likely to have visited any garden where pesticides were sprayed in the 3 days preceding the illness onset compared with the controls. In the neighborhood analysis, cases were 8.4 times (29% versus 5%, aOR = 8.4, 95% CI = 1.4–49.9) more likely to have visited any garden that used pesticides in the 3 days preceding the illness onset compared with the controls. Eating lychees within 24 hours prior to onset of illness was not associated with developing illness among cases compared with controls from villages (57% versus 71%, aOR = 0.6, 95% CI = 0.1–2.1) or neighborhoods (57% versus 61%, aOR = 0.9, 95% CI = 0.2–2.9). Eating mangoes in the 24 hours preceding the illness onset was protective in the village-level case–control analysis (43% versus 85%, aOR = 0.1, 95% CI = 0.0–0.6). In the both sets of case–control analysis, “control respondents were significantly more likely to report not knowing if their child had the exposure in question, compared with the cases (Supplemental Tables 1 and 2).”

### Qualitative study.

#### Exposures of the case-patients.

Lychees are commercially grown in the affected subdistricts and these subdistricts are well known for their widespread cultivation of lychees. There were 15 lychee orchards adjacent to or nearby 13 case-patients’ households. Fifty-seven percent (8/14) of the case-households were adjacent to at least one lychee orchard and five (36%) of the others were within 10 m of the orchards. Family members of one case-household informed us that there were no lychee orchards nearby the case-patient’s house and the case-patient did not visit any lychee orchards within 7 days prior to his illness. Family caregivers of all case-patients mentioned that the case-patients usually played in their household premises. Eighty-five percent of the case-patients visited lychee orchards within 3 days of illness onset and the average elapsed time between the most recent visit to a lychee orchard and illness onset was 16.5 hours. The family members of the children reported that they did not always pay close attention to what children were eating or doing in the orchards, including in the 24 hours before onset of illness. However, family members of eight case-patients recalled that the eight case-patients had consumed lychees from the nearby orchards in the 24 hours prior to onset of illness.

The orchard caretakers mentioned that due to pests and a physiological disorder in the lychee trees, many lychees had cracked and dropped on the ground in the orchard. Case-patients or their family members often visited lychee orchards and collected dropped lychees from the ground and ate and/or fed them to case-patients. The family caregivers mentioned that people in their community did not wash lychees before eating them and the children usually used their teeth to peel them before eating them. The children ate the flesh part of the lychee and discarded the seeds and peel.

The community members of the affected villages informed us that wholesalers from around the country leased the lychee orchards for multiple years at a time. Orchard leaseholders explained that they frequently hired residents from the community to work as orchard caretakers. Eight of the 14 case-patients lived with someone who worked in lychee orchards and three of these family members were also involved in spraying pesticides in the orchards during the outbreak period. While visiting case-patient households, we found equipment used to apply pesticides in two households and pesticides in a bottle and in a packet in one household. The community members also informed us that lychee orchard caretakers often had children assist with fruit harvesting. One case-patient was involved in harvesting lychees. A neighbor of the case mentioned that,

The lychee businesspersons hired children for collecting fruits from small lychee trees. Since the trees are small, the children can easily go to the small branches and collect lychees. The labor costs of children are also less expensive.

#### Use of agrochemicals in lychee orchards.

**Figure 2.**
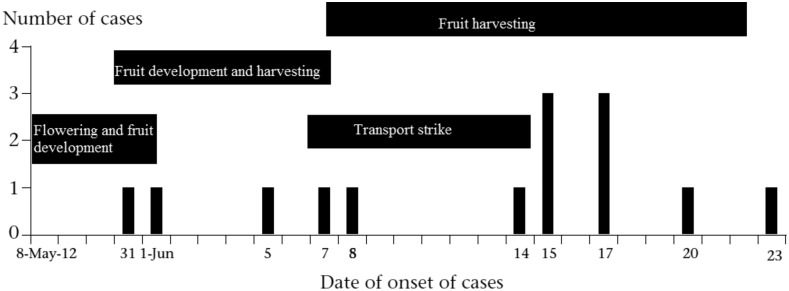
Distribution of cases and timeline of lychee production and harvesting, northern Bangladesh, 2012.

The orchard caretakers explained that the duration from lychee flowering to fruit harvesting is approximately 1 month starting from May to June (Figure 2), though the timeline of lychee harvesting could vary based on the lychee variety. The caretakers described using different types of agrochemicals at different stages of lychee flowering, fruit maturation, and harvesting. The caretakers explained that lychee orchard wholesalers bought pesticides from local pesticide dealers as well as from unregistered sellers near the Indian border. Before providing the pesticides to the caretakers, the wholesalers sometimes took the label off of the bottles or bags of agrochemicals. The caretakers said that the wholesalers did so because they did not want orchard caretakers and local gardeners to know what pesticides they were using.

Caretakers reported that they sprayed different vitamins and fertilizers (e.g., boron) on the trees before flowering. However, the caretakers could not recall the names of the vitamins. After flowering and during fruiting, the orchard caretakers reportedly used several insecticides, including cypermethrin, endosulfan, alpha-cypermethrin, and lambda-cyhalothrin. The mother of one case-patient mentioned that the smell of pesticides sprayed in the neighboring orchard was so strong that it was difficult for them to stay at home after the chemicals had been sprayed and that the smell persisted for several hours. During fruit maturation, the orchard caretakers used different fungicides along with insecticides to protect young lychees from insects, fungus, and the sun. The orchard caretakers also reported using plant growth regulators to improve growth, protect flowers, and prevent fruit from dropping. As fruit matured, they continued spraying insecticides, fungicides, and plant growth hormones two to three times a week. Caretakers mentioned they used hair shampoo on lychees to avoid spotting or remove spots from the fruit. Caregivers also reportedly used carbendazim (benzimidazole carbamate fungicide) to enhance lychee coloration. The caretakers said that they discarded the used bottles and packets of pesticides in the orchards. The team visited four orchards in three case-patient’s villages and collected empty pesticide, insecticide, fungicide, and other chemical bottles and packets that had been discarded in lychee orchards. The active ingredients listed on the bottle and packet labels included pyrethroids, endosulfan, organochlorine, dithiocarbamates, benzimidazole, triazole, soluble boron, and soluble potash.

The orchard caretakers we interviewed supplied lychees to different areas of the country. Their distribution was disrupted by a 5-day nationwide strike among bus and truck drivers during the second week of June 2012. The caretakers mentioned that this was the peak week for harvesting lychees, but they did not harvest the fruits, as there was no alternative way to transport the lychees to markets around the country. Orchard caretakers from two communities informed us that they used carbendazim almost every day during the strike to delay the fruit from ripening and minimize their financial losses from the transport strike.

## DISCUSSION

The clinical manifestations in case-patients including sudden onset without a prodromal phase, becoming unconscious within a median of 2.5 hours of illness onset, and the short duration between onset of illness to death all suggest that the outbreak was more likely due to a toxic poisoning than an infection. The outbreak was restricted to the lychee season and to a geographic area in which lychees are grown, which suggests that this outbreak of AES may be linked to exposure to lychees or lychee orchards.^12^

Our epidemiological study identified a strong association between illness and exposures to lychee orchards and lychee orchard workers in the village-level case–control analysis, providing evidence that this outbreak was related to an exposure in lychee orchards, similar to findings reported from similar outbreaks of AES in India.^6,12–14,17,24^ The elevated odds in both nearby village level and neighborhood control analysis suggest visiting a lychee orchard prior to illness onset, visiting a garden where pesticides were sprayed, and family members working in lychee orchard increase the risk of illness among children.^6^ The onset of this outbreak during lychee cultivation and the increasing number of cases during fruit harvesting time further supports the evidence of lychee-associated illness. Moreover, the recent outbreak of AES among children in 2015 in the same lychee production area of Dinajpur District further strengthens our assertion that the outbreak of AES among children may relate to some kind of behaviors and practices around lychee cultivation in the affected area.^18^

In India, the leading hypothesis of AES etiology among children living near lychee orchards was the ingestion of phytotoxins, specifically methylenecyclopropyl-glycine that is present in lychee seeds and pulp, which can lead to hypoglycemia among malnourished children.^6,25^ Without acute biological specimens, and therefore, lack of measured blood glucose levels from children in this outbreak in Bangladesh, we were unable to investigate this hypothesis directly. However, our epidemiologic and clinical findings suggest that methylenecyclopropyl-glycine alone may not explain this AES outbreak among children. There are multiple lines of evidence that agrochemical poisoning may also have contributed.

First, lychee consumption was not statistically significantly associated with child illness in our case–control analysis, which is apparently consistent with the community controls in India.^17^ Lychees are cultivated, harvested, and consumed in different parts of Bangladesh^26^; however, the lychee-associated outbreaks have been reported only in Dinajpur and Thakurgaon. If lychee consumption were the cause, we would expect cases to be reported from other parts of Bangladesh, which is also consistent with the findings in India and Vietnam where outbreaks were clustered in a few lychee-producing areas.

Second, visiting any garden that recently used pesticides increased the risk of developing illness in the neighborhood and village-level analysis. The qualitative investigation identified heavy use of multiple, highly toxic agrochemicals in lychee orchards. Packets of endosulfan, an organochlorine insecticide banned due to the deleterious health effects in more than 80 countries, were observed in the lychee orchards suggesting recent use of this highly toxic pesticide.^27^ In most fruits, the half-life of endosulfan is 3.7–4.6 days.^28^ Endosulfan-related mortality and morbidity have been reported from different parts of the world including India, Turkey, and Bangladesh.^29–31^ Since 1978, the local government of Kerala, India, estimated that indiscriminate use of endosulfan caused poisoning among 4,270 humans including 500 deaths.^30^ The frequent use of multiple agrochemicals in the lychee orchards increases pesticide exposures among children in several ways.^32^ In these outbreak communities, affected children and their family caregivers collected cracked and dropped lychees from the orchard or bought from a local market. During spraying pesticides over lychee trees, the edible fruit fleshes of cracked lychees were likely exposed to pesticides. Since the children reportedly did not wash the lychees before eating and used their teeth to peel the lychees, unwashed lychees contaminated with pesticides may have increased the risk of poisoning.^33^

In addition, the children were exposed to these chemicals through multiple routes beyond just lychee consumption. Their frequent exposure to lychee orchards suggests that they could have been exposed to pesticides there, including through skin contact, ingestion, and perhaps inhalation. Family members of the case-patients who worked in the lychee orchards or were involved in pesticide spraying in the orchards might have brought pesticides into their homes in the form of residues on their tools, clothes, shoes, and skin and children might have also been exposed to pesticides through contact with these family members and their items.^34^ Children are more susceptible to pesticide poisoning than adults in a contaminated environment because they eat, drink, and breathe more per unit of body weight.^35^ In addition, while playing in their household premises or in the orchard, children are more likely to put their fingers and other objects that might be contaminated with pesticides into their mouths, including bottles, clothes, and mud.^36^

Third, children involved in this outbreak presented with clinical illnesses similar to those identified during confirmed outbreaks of pesticide poisoning in Bangladesh.^37^ During an outbreak of sudden child deaths in 2009, investigators detected carbamate insecticides in the biological specimens collected within 6 hours of illness onset, which suggested poisoning.^37^ Similarities between that outbreak and those cases include age distribution of the case-patients, a sudden outcry, onset of illness early in the morning, mid-dilated or fixed pupils, lung crepitations on auscultation, excess sweating, froth at the mouth, excess respiratory secretions, convulsions, and coma.^37^ In our study, the signs and symptoms are also consistent with acute pesticide poisoning in children reported in different countries of the world.^38–41^ Fever has been reported as part of the clinical syndrome following pesticide poisoning in many countries.^42^ Although family members of four case-patients reported fever, it was not the initial symptoms for them, and was not confirmed in any of the clinical records. The onset of illness of the case-patients was distributed throughout the day. Studies showed that the time of occurrence of symptoms and signs depends on the route of exposures, poison load, chemical nature, and solubility characters of the compound.^43^ For acute pesticide poisoning, the signs and symptoms may occur after minutes to 24 hours of exposures, thus supporting the different timeline of the onset of illness among case-patients.^43^

Fourth, the outbreak ended following the onset of the monsoon rains, which suggest that the toxin associated with the lychees was on their surface, rather than contained in the fruit. In India, a similar pattern was observed, where the number of cases peaked in mid-June and declined immediately after the onset of monsoon rains.^6^ Moreover, the findings of washing fruits and vegetables protective against lychee-associated illness in India further supports our assertion that toxin associated with the lychees may be on the surface.^17^

Diagnosing unintentional pesticide or other agrochemical poisoning among children is difficult.^44^ The signs and symptoms of agrochemicals poisoning may vary by type of chemicals, route of exposures, and age of case-patients.^41,45^ In our study, most of the case-patients presented with reduced levels of consciousness or seizures rather than the classic nicotinic and muscarinic effects often seen in adults. This observation is consistent with previous findings of pesticide poisoning in young children, where the predominant manifestations were neurological.^41,44–46^ In addition, pesticide poisoning may imitate other severe disorders such as heat stroke, pneumonia, asthma, hypoglycemia, or intestinal secretions.^47^ Acute exposures to organophosphate and carbamate pesticides in children accumulate acetylcholine at the autonomic ganglia resulting in hypertension, pallor, and hypoglycemia.^48^ This could explain why most of the case-patients fall ill between 2 am and 8 am when blood glucose reaches to its lowest levels.^49^

Confirming a diagnosis of toxic poisoning requires acute specimens and is aided by good clinical records. Since case-patients were admitted in serious condition and died quickly after arriving at the hospital, attending physicians did not collect acute specimens from patients during this outbreak, making a confirmed determination of the cause of death in these patients impossible. The clinical files for some case-patients were either missing or incomplete; so much of what we know about the clinical illness comes from interviews with the family caregivers. Case ascertainment was limited to admitted children at Dinajpur Medical College Hospital; therefore, we might have missed cases from villages whose residents might seek care in other facilities or who died before reaching the hospital. In addition, we missed adult cases who might have exposed to pesticide and developed mild symptoms but were not hospitalized as our case finding was limited to children with severe illness in the hospital. Moreover, lack of access to health facilities and cultural practices that favor health seeking from a traditional healer before consulting a trained physician limit the detection and reporting of AES cases in the affected areas.

A second limitation was that orchard caretakers often received pesticides from the lychee orchard leaseholders without any labels, so the orchard caretakers were not fully aware of the pesticides they applied to the lychee trees. Therefore, we were unable to identify all the pesticides used in affected villages and compared with the control villages. Although we identified the names of the agrochemicals used in the orchards by visiting the lychee orchards and collecting empty pesticide containers and bags from the orchards, we were unable to confirm the timeline of pesticides used in the orchards from the discarded pesticide bottles and packets.

A third limitation of this study was limited power due to the small number of cases. We aimed to identify as many risk factors for child death as possible and therefore, we did not adjust the *P* values for multiple comparisons so that potential risk factors would not be eliminated. Nonetheless, our findings can help identifying several possible risk factors and guide future studies for clear understanding of these repeated outbreaks and sudden deaths in Bangladesh and in countries that experienced similar outbreaks in south Asia.

A fourth limitation was ascertaining illness history, especially among the 13 case-patients who died. We relied on case-patients’ family caregivers and neighbors to recall and report the sign and symptoms of the case-patients. We used medical records to supplement case-patients reports of illness history, but often these records were not available for all case-patients. However, the qualitative investigation began during the outbreak, which provided for better recall of illness history and time line of events.

In addition, proxies of both cases and controls noted that many of the child activities generally occur outdoors and, therefore, they were unable to recall exposure histories. Moreover, proxies of case-patients might have reflected more about exposures and identified exposures more thoroughly than controls; our analysis showed that controls had missing exposure information significantly more often than cases. The observed protective association of eating mangoes and developing illness might be due to the large differences in missing information between cases and controls. However, if all of our findings were due to recall bias, we would expect more spurious associations rather than a pattern of associations consistent with pesticide poisoning. For instance, although there were many exposures that cases recalled significantly more frequently than controls, only exposures related to lychee orchards or other gardens where pesticides were applied were associated with illness. In addition, although there was no significant difference in recall between cases and controls regarding visiting a lychee orchard within 24 hours of illness or having family members work in lychee orchards, those were also significantly associated with illness in the nearby village-level analysis.

In India, Vietnam, and Bangladesh, the most common finding of outbreaks of AES occurring during the lychee harvesting season is a spatial relationship to lychee orchards. Since outbreaks of illness associated with lychee and lychee orchards have repeatedly been reported from these countries in Asia, we recommend standardizing protocols across these countries to allow for comparisons between the resulting outbreak investigations. Moreover, future studies should target establishing surveillance to detect AES clusters in lychee producing areas, collect acute blood and urine specimens of case-patients, and provide a time line of symptom onset along with records of different agrochemicals used to help elucidate a common thread among countries as well as potential risk factors for illness among children.

From our experience of investigating the AES outbreak, collecting information about pesticide exposures only through questionnaires administered in the hospital may be insufficient to understand the context where the outbreak occurred. Indeed, communities are often unaware of contact they have had with agrochemicals. Therefore, we recommend qualitative exploration along with the surveillance to explicate local perceptions, behaviors, and practices that may have contributed to the outbreak occurrence, particularly those related to community use of toxic chemicals. We also recommend making an inventory of agrochemicals used on the lychee orchards, collecting samples of agrochemicals along with environmental samples, and testing them to identify the implicated chemicals and or toxins. In Bangladesh, pesticide regulation may be difficult due to lack of monitoring and pesticide residue analysis^50^ as well as the more general difficulty that weak states face in enforcing environmental regulations. Educating farmers on pesticide handling including proper storage, use of aprons and gloves or washing hands and cloths with soap after pesticide spraying might reduce children’s pesticide exposures at their dwelling place.

Finally, in India, mortality decline among AES case-patients after rapid correction of hypoglycemia with intravenous dextrose. Although we were unable to measure blood glucose level among the case-patients, however, distribution of blood glucose meter among clinician may help identifying patients with hypoglycemia and rapid correction of hypoglycemia with intravenous glucose may reduce mortality in children in Bangladesh.

## Supplementary Material

Supplemental Table.
