# Supplementary material for: Outbreak of Sudden Death with Acute Encephalitis Syndrome Among Children Associated with Exposure to Lychee Orchards in Northern Bangladesh, 2012
Source: Am J Trop Med Hyg. 2017 Jul 24;97(3):949–57. doi: 10.4269/ajtmh.16-0856 (PMC5590581; doi:10.4269/ajtmh.16-0856)
Supplement: Supplementary file 1 [file tpmd160856.SD1.pdf]

Supplemental Table 1

Proportion of cases with missing exposure information compared with nearby village controls, northern Bangladesh, 2012

Exposures	Case *n/N* (%)	Control *n/N* (%)	*P* value
Exposures in the 24 hours preceding illness onset of case-patients			
Food			
Tube well water	0/14 (0.0)	1/56 (1.8)	0.614
Lychee	0/14 (0.0)	25/56 (45)	0.002
Mango	0/14 (0.0)	30/56 (54)	< 0.001
Papaya	0/14 (0.0)	21/56 (38)	0.006
Potato	0/14 (0.0)	14/56 (25)	0.036
Ladies finger	0/14 (0.0)	28/56 (50)	< 0.001
Egg plant	0/14 (0.0)	36/56 (64)	< 0.001
Tomato	0/14 (0.0)	28/56 (50)	< 0.001
Banana	0/14 (0.0)	30/56 (54)	< 0.001
Bitter gourd	0/14 (0.0)	24/56 (43)	< 0.003
Corn	1/14 (7.1)	21/56 (38)	0.028
Lentils	0/14 (0.0)	34/56 (61)	< 0.001
Environment			
Visiting lychee orchard	3/14 (21)	19/56 (34)	0.367
Visiting a mango orchard	1/14 (7.1)	16/56 (29)	< 0.094
Visiting a vegetable garden	0/14 (0.0)	2/56 (3.6)	0.473
Visiting an orchard that leased for commercial production	0/14 (0.0)	1/56 (1.8)	0.614
Exposures in the 3 days preceding illness onset of case-patients			
Visiting a lychee orchard	1/14 (7.1)	6/56 (11)	0.690
Visiting a mango orchard	1/14 (7.1)	8/56 (14)	0.474
Visiting a vegetable garden	0/14 (0.0)	6/56 (11)	0.200
Visiting any garden that sprayed pesticides	0/14 (0.0)	5/56 (8.9)	0.245
Plucked lychees from the orchard	1/14 (7.1)	3/56 (5.4)	0.797
Plucked mangoes from the orchard	0/14 (0.0)	2/56 (3.6)	0.473
Visiting any garden while pesticides were being applied	1/14 (7.1)	13/56 (23)	0.178
Other exposures			
Having lychee orchard adjacent to the house	0/14 (0.0)	0/56 (0.0)	Undefined
Having mango orchard adjacent to the house	0/14 (0.0)	0/56 (0.0)	Undefined
Having vegetable garden adjacent to the house	1/14 (7.1)	2/56 (3.6)	0.555
Family members work in lychee orchard	0/14 (0.0)	3/56 (5.4)	0.375
Family members work in mango orchard	0/14 (0.0)	2/56 (3.6)	0.473
Family members work in vegetable garden	0/14 (0.0)	1/56 (1.8)	0.614
Family members with occupation in agriculture	1/14 (7.1)	5/56 (8.9)	0.830
History of animal or insect bite	0/14 (0.0)	2/56 (3.6)	0.473

Supplemental Table 2

Proportion of cases with missing exposure information compared with neighborhood controls, northern Bangladesh, 2012

Exposures	Case *n*/*N* (%)	Control *n*/*N* (%)	*P* value
Exposures in the 24 hours preceding illness onset of case-patients			
Food			
Tube well water	0/14 (0.0)	0/56 (0.0)	Undefined
Lychee	0/14 (0.0)	12/56 (21)	0.057
Mango	0/14 (0.0)	15/56 (27)	0.029
Papaya	0/14 (0.0)	16/56 (29)	0.022
Potato	0/14 (0.0)	11/56 (20)	0.070
Ladies finger	0/14 (0.0)	18/56 (32)	0.013
Egg plant	0/14 (0.0)	17/56 (30)	0.017
Tomato	0/14 (0.0)	15/56 (27)	0.029
Banana	0/14 (0.0)	16/56 (29)	0.022
Bitter guard	0/14 (0.0)	13/56 (23)	0.046
Corn	1/14 (7.1)	14/56 (25)	0.145
Lentils	0/14 (0.0)	18/56 (32)	0.013
Environment			
Visiting lychee orchard	3/14 (21)	19/56 (34)	0.368
Visiting a mango orchard	1/14 (7.1)	12/56 (21)	0.218
Visiting a vegetable garden	0/14 (0.0)	2/56 (3.6)	0.473
Visiting an orchard that leased for commercial production	0/14 (0.0)	2/56 (3.6)	0.473
Exposures in the 3 days preceding illness onset of case-patients			
Visiting a lychee orchard	1/14 (7.1)	0/56 (0.0)	0.044
Visiting a mango orchard	1/14 (7.1)	2/56 (3.6)	0.555
Visiting a vegetable garden	0/14 (0.0)	9/56 (16)	0.108
Visiting any garden that sprayed pesticides	0/14 (0.0)	0/56 (0.0)	Undefined
Plucked lychees from the orchard	1/14 (7.1)	1/56 (1.8)	0.283
Plucked mangoes from the orchard	0/14 (0.0)	0/56 (0.0)	Undefined
Visiting any garden while pesticides were being applied	1/14 (7.1)	6/56 (11)	0.690
Other exposures			
Having lychee orchard adjacent to the house	0/14 (0.0)	0/56 (0.0)	Undefined
Having mango orchard adjacent to the house	0/14 (0.0)	0/56 (0.0)	Undefined
Having vegetable garden adjacent to the house	1/14 (7.1)	1/56 (1.8)	0.283
Family members work in lychee orchard	0/14 (0.0)	2/56 (3.6)	0.473
Family members work in mango orchard	0/14 (0.0)	0/56 (0.0)	Undefined
Family members work in vegetable garden	0/14 (0.0)	2/56 (3.6)	0.473
Family members with occupation in agriculture	1/14 (7.1)	3/56 (5.4)	0.797
History of animal or insect bite	0/14 (0.0)	2/56 (3.6)	0.473
